# Program Practices Predict Intergenerational Interaction Among Children and Adults

**DOI:** 10.1093/geront/gnab161

**Published:** 2021-11-06

**Authors:** Shannon E Jarrott, Shelbie G Turner, Jill Juris, Rachel M Scrivano, Raven H Weaver

**Affiliations:** 1 College of Social Work, The Ohio State University, Columbus, Ohio, USA; 2 College of Public Health and Human Sciences, Oregon State University, Corvallis, Oregon, USA; 3 Beaver College of Health Sciences, Appalachian State University, Boone, North Carolina, USA; 4 Department of Human Development, Washington State University, Pullman, Washington, USA

**Keywords:** Adult day services, Behavioral outcome, Intergenerational program, Intergenerational relationship, Multilevel model

## Abstract

**Background and Objectives:**

Intergenerational programs, those engaging youth and adults of nonadjacent generations in shared programming for mutual benefit, are attracting increasing attention from funders, policymakers, and practitioners for the range of goals they can support. The mechanisms by which these goals are achieved are rarely studied. To address this gap, we analyzed the associations between specific intergenerational implementation practices and younger and older participant outcomes.

**Research Design and Methods:**

Activity leaders at 5 sites serving adults and preschoolers received training to implement 14 evidence-based practices during intergenerational activities involving 84 adults (*M* = 75.25 years; range = 55–98) and 105 preschool participants (*M* = 3.26 years; range = 2–5) over 4 years. Measures of activity leaders’ implementation of these practices and participants’ behavioral responses to programming were gathered. We utilized multilevel modeling to test whether variations in implementation of practices were associated with variations in participants’ responses to programming on a session-by-session basis.

**Results:**

For both preschool and adult participants, analyses revealed that the implementation of certain practices was associated with significantly more intergenerational interaction. When more practices were implemented reflecting factors of (a) participant pairing and (b) person-centered care, both child and adult intergenerational interactions were higher.

**Discussion and Implications:**

Practices used by intergenerational activity leaders during programming help to explain within-person responses of both child and adult participants. Intergenerational relationships may be a powerful means to achieve diverse goals; they depend on skillful practice by trained activity leaders.

Nonfamilial intergenerational programs engage younger and older people belonging to nonadjacent generations (i.e., youth 24 years of age and younger and adults 50 years of age and older; Generations United, 2018) in shared programming for mutual benefit. A common program model relies on university students to complete service learning at a senior center, or virtually, during routine visits over several months. For example, the Cyber-Seniors program engages interested older adults to build their technology skills and participating students to learn about the diversities of old age and build self-efficacy (Leedahl et al., 2019). Another common model for intergenerational programming involves co-located care programs, also known as shared sites, which usually involve early childhood and adult day services (ADS) or residential care programs. Children and older adults who join varied shared programming in this type of setting have benefited from enhanced empathy among the children (Femia et al., 2008) and active engagement among frail older adults (Kamei et al., 2021).

Interest in intergenerational programs and associated evaluation methods has been growing. Practitioners responding to a 2018 national survey indicated a need for greater program implementation and evaluation resources (Jarrott & Lee, in press). Researchers continue to attend to mechanisms by which intergenerational program outcomes are achieved, including elements of the physical environment and implementation strategies that promote intergenerational interaction (Bunting & Lax, 2019; Weaver et al., 2019). Funders such as The Eisner Foundation (https://eisnerfoundation.org) and RRF: Foundation for Aging (https://www.rrf.org) have earmarked funds for applications using intergenerational strategies. Finally, the unanimously approved reauthorization of the Older Americans Act (Supporting Older Americans Act, 2020) incorporates new language encouraging and prioritizing the delivery of intergenerational services.

With growing emphasis on the potential benefits of intergenerational strategies and programming, it is imperative to know their effects on participant outcomes. Researchers must also study the means by which these outcomes are achieved. To work toward that goal, we analyzed the associations between the implementation of intergenerational practices and younger and older participant outcomes. Activity leaders delivering intergenerational programming were trained to use practices informed by theories of contact (Pettigrew, 1998) and personhood (Kitwood, 1997), which support positive exchange between disparate groups by offering stakeholder-endorsed, strengths-based programming that fosters relationships. Programming content varied to reflect developmental goals and interests of participants, as it commonly does at intergenerational programs, frequently exercising physical, artistic, literacy, musical, and social skills. Data captured the practices used by leaders and child and adult participants’ responses during programming.

## Literature Review

Intergenerational programs achieve a range of population-specific goals. Experience Corps, which trains volunteers 50 years of age and older to help children achieve grade-level reading skills in 21 cities across the United States, offers some examples (Varma et al., 2015). Volunteers’ perceptions of achieved generativity and elementary school students’ objectively measured reading level demonstrated significant improvement; greater benefits were associated with higher levels of participation (Gruenewald et al., 2016). In other studies, university service-learning students engaged in topical conversations with older residents of long-term care facilities demonstrated improved confidence in their public speaking skills (Bunting & Lax, 2019) and reduced ageism (Andreoletti & Howard, 2018). Older conversation partners experienced increased cognitive stimulation, empowerment (Bunting & Lax, 2019), and a sense of generativity (Andreoletti & Howard, 2018). Young and older participants recognized their shared humanity and expressed a greater appreciation for one another (Andreoletti & Howard, 2018).

Evidence of the impact of intergenerational programs is almost exclusively positive (Galbraith et al., 2015; Jarrott, 2011; Lee et al., 2020), but challenges still exist. For example, staff working at shared sites who were interviewed about experiences with their intergenerational program described challenges building an effective partnership with collaborators due to scheduling, staffing requirements, and philosophical differences (Weaver et al., 2019). Experience Corps volunteers described feeling that their talents were underutilized when teachers were slow to give them meaningful roles in the classroom (Varma et al., 2015). Encountering challenges such as securing participants, program funding, and evidence of short- and long-term impacts results in some intergenerational programs discontinuing shared programming (Jarrott & Lee, in press). Investigations rarely consider the role of implementation factors (Jarrott, Scrivano et al., 2021), which limits the potential for program tailoring or replication (Powell et al., 2019). Despite these challenges, research has identified evidence- and practice-informed strategies to develop successful intergenerational programming.

Evidence-based intergenerational practices were presented by Jarrott et al. (2019), representing a practice model developed over several years at a shared site providing intergenerational care located in a university setting. These practices were incorporated into multimodal training materials for community-based practitioners in a multiyear study of shared preschool and adult services and programming. Resultant data informed a series of studies focused on the practice model, culminating in the current article. In one study, investigators compared activity leaders’ self-report of practice implementation during intergenerational activities with trained observers’ coding of practice implementation of the same activities, determining that activity leaders consistently implemented most of the recommended practices (Juckett et al., 2021). Variability in the frequency of each practice being implemented led Juckett et al. to suggest that greater fidelity might be achieved with modifications to the model or its delivery method. An exploratory factor analysis of the same observational data of activity leaders’ implementation of these practices during intergenerational programming illustrated three dimensions of practice: (a) promoting participant pairing, (b) environment-centered strategies, and (c) staff knowledge of participants (Jarrott, Turner et al., 2021). These dimensions may prove critical to the practice model and should be tested for their association with program participant outcomes.

Practices that emerged from this line of inquiry by Jarrott et al. (2019; Jarrott, Turner et al., 2021; Juckett et al., 2021), such as attending to environmental features and incorporating participants’ cultures and experiences into programming, were echoed in a systematic scoping review of practices that influence measured intergenerational program outcomes (Jarrott, Scrivano et al., 2021). For example, practices related to offering meaningful, age-appropriate roles and incorporating mechanisms of friendship among intergenerational participants were represented across several studies. Frequently, these practices were identified in the process of analyzing qualitative data (Alcock et al., 2011); that is, they were not specified a priori in a model of change. A few investigators conducted quantitative analyses to identify specific practices associated with outcomes. For instance, analysis of Experience Corps student participant outcomes revealed greater benefits among students with higher levels of participation in Experience Corps (Gruenewald et al., 2016), leading authors to emphasize the practice of frequent, regular participation in the tutoring program.

Even small pilot projects have tested the association between specific practices and participant experiences with an intergenerational program (Gonzales et al., 2010). Contact theory, with tenets of authority support, equal group status, cooperation, a common goal, and mechanisms of friendship (Allport, 1954; Pettigrew, 1998), led Gonzales et al. (2010) to implement an arts-based intergenerational program involving medical students and older adults, with a goal of improving students’ attitudes toward older adults and increasing interest in working with an aging population. Arts-focused programming was chosen to support the contact theory tenet of *supporting equal group status among contact members*; because most younger and older participants lacked artistic expertise, they shared a *novice* status during the program. Qualitative data analysis revealed a sense of being equals that was perceived as enhancing the experience for younger and older participants.

The current study adds to a small but essential body of work exploring the link between specific implementation practices and outcomes of intergenerational programming. We address the relationship between activity leaders’ use of specific evidence-based practices and behavioral responses of young child and adult participants. As intergenerational programs figure more centrally in public interest, practice, and policy (Jarrott & Lee, in press) the mechanisms by which they achieve their goals are essential for replication, adaptation, scaling up, and sustainability (Pettigrew & Tropp, 2008; Powell et al., 2019).

We take an intraindividual variability approach that captures within-participant differences to analyze how the implementation of intergenerational programming Best Practices (BPs) affects participants’ intergenerational interactions on a session-by-session basis. We present an analysis of within-participant differences as we expect participant responses to fluctuate (Hoffman & Stawski, 2009) as activity leaders vary in the extent to which they implement the BPs within each session. Thus, based on extant literature (Heydon et al., 2017), we hypothesize that certain factors—in this study, BPs on Jarrott et al.’s BP Checklist—can alter intergenerational interaction session-by-session. Our research questions and hypotheses are as follows:

***Research Question 1 (child)*** *: Is the use of BPs associated with the extent to which a child engages in intergenerational interaction at the within-person level?*

We hypothesized that when a child participated in a session that had higher BP implementation than the average BP implementation of all that child’s sessions, that child would have higher intergenerational interaction for that session than their average interaction across their sessions.

***Research Question 2 (adult)*** *: Is the use of BPs associated with the extent to which an adult engages in intergenerational interaction at the within-person level?*

We hypothesized that when an adult participated in a session that had higher BP implementation than the average BP implementation of all that adult’s sessions, that adult would have higher intergenerational interaction for that session than their average interaction across their sessions.

## Method

### Study Procedure

We analyzed data from a United States Department of Agriculture; Children Youth and Families at Risk Sustainable Community Project grant entitled Project TRIP (Transforming Relationships through Intergenerational Programs). The goal of Project TRIP was to build community with evidence-based practices by increasing and improving relationships between younger and older participants, enhancing connections among community members, and increasing sustainability of intergenerational programs. Project TRIP trained child and adult care staff to deliver intergenerational programming between 2011 and 2016 at five sites in Virginia that included three co-located ADS (serving adults aged 18 and older needing ongoing care and supervision) and childcare centers (serving preschool-aged children between 2 and 5 years old). Another co-located site consisted of ADS, a senior center, and a childcare center. At the fifth site, community-dwelling adults volunteered to join programming with children at the childcare center. Our research has demonstrated that older adults with diverse physical and cognitive abilities, including advanced dementia, can engage actively and appropriately in intergenerational programming (Jarrott & Bruno, 2003; Jarrott & Smith, 2011) if staff utilize theory- and evidence-informed practices. Thus, we did not expect ADS participants to engage differently than senior center participants or adult volunteers from the community.

Intergenerational sessions led by the trained staff were offered at least once per week at each site and usually lasted approximately 20–30 minutes, with activities ranging from creative movement to crafts to scientific inquiry. Activity leaders participated in a multifaceted training focused on intergenerational BPs (Juckett et al., 2021) before offering intergenerational programming. The training encouraged activity leaders to appropriately integrate the 14 intergenerational BPs during subsequent intergenerational programming, which was expected to vary in content. The method of delivering this training was modified mid-way through the study, shifting from a day-long shared training session to self-paced online training; follow-up training on intergenerational topics of interest was delivered in-person throughout the study. To learn more about the initial 14 BPs, see the work of Jarrott et al. (2019); for findings related to staff perspectives on training initiatives, see the work of Weaver et al. (2019). All research activities were approved by the Virginia Tech Institutional Review Board (IRB; #11-580).

### Data Collection

Quantitative observations of children and adults participating in intergenerational programs were collected for review by trained coders. Although intergenerational programming at the five sites served multiple goals (i.e., solitary engagement, turn-taking, active watching, and interacting), data included in the current analysis represent those sessions designed to support intergenerational interaction. Data for sessions that did not have a goal of fostering interaction were excluded, such as the session when a visiting musician performed and active watching was the intended behavior of participants. Though all the activities shared a goal of supporting intergenerational interaction, programming content varied, including gardening, cooking, arts, music, and physical activities, among others. Such variety is common in child and adult care programs, including those offering intergenerational programming (Jarrott & Lee, in press). Given the focus on intergenerational relationships, adult participants younger than 50, who may be served by programs like ADS for a variety of care needs, were excluded from the analysis. We included participants who had at least three 15-s time frames (representing approximately 7 min of participation), in which they were observed in turn with up to five other participants, yielding a final analytic sample of 225 observations of 84 adults and 266 observations of 105 children.

Over a 4-year period, observations took place in-person at three sites and through video-recordings at two sites that were geographically remote. The video-recorded intergenerational sessions were uploaded to a secure, IRB-approved storage system. To document intergenerational interaction, seven individuals completed a four-step training process to become reliable observers of the intergenerational observation scale (IOS). The IOS was developed by Jarrott et al. (2008), based on the Play Observation scale from Rubin (2001) that evaluated social behavior of play; it has demonstrated acceptable reliability (Jarrott et al., 2008) and a positive association between activity leader practices and level of intergenerational interaction (Jarrott & Smith, 2011). In the current study, observers achieved acceptable interrater reliability using the IOS with Cohen’s *k* ranging from 0.64 to 0.75 (Landis & Koch, 1977). Observers also completed a BP Checklist for each intergenerational activity (Table 1), which has demonstrated high levels of interrater agreement (Juckett et al., 2021). The coded observations using the IOS and BP Checklist are the data for this study.

**Table 1.** Best Practice Checklist Items, by Factor, and Percentage Implemented

	Percentage implemented	
Best practice item	Children	Adults
Factor 1: Promoting participant pairing		
4. Ratio of adult to child participants was equal or near equal	85%	81%
5. Seating arrangement used intergenerational pairs or small groups	89%	88%
6. Materials were paired	69%	72%
Factor 2: Person-centered strategies		
1. Activity leaders discussed the activity in relation to participant interests or experiences to encourage intergenerational interaction	55%	56%
2. The activity was age- and role-appropriate for child participants	99%	99%
3. The activity was age- and role-appropriate for adult participants	88%	88%
7. Activity leaders guided the activity to promote intergenerational interaction	66%	70%
11. Staff avoided overfacilitation	83%	84%
14. The intergenerational programming session was documented (e.g., photos were taken or evaluation forms completed)	80%	81%
Factor 3: Socioemotional accommodations (referred to as staff knowledge of participants in Jarrott, Turner et al., 2021)		
9. Adaptations to physical space	80%	78%
10. Distractions were minimized	79%	78%
12. Activity leaders were responsive to both generations of participants	96%	94%
13. Activity leaders moved around the activity area	100%	100%

Analyzing whether implementation of these practices facilitates more intergenerational interaction can offer justification for their use in intergenerational programming and provide evidence-based guiding principles for activity leaders. In this study, we analyze how BPs grouped together into the three factors identified by Jarrott, Turner et al. (2021) are associated with a greater session-by-session percentage of intergenerational participation among child and adult participants, as indicated through the IOS.

### Measures

#### Intergenerational observation scale (outcome)

Through structured observations, trained observers record child and adult participants’ behavioral responses to the intergenerational program. The IOS divides each intergenerational session into six 30-s coding intervals. For each coding interval, observers indicate individual participants’ predominant behavior from six possible options: (a) interacting intergenerationally, (b) interacting with a similar-age peer, (c) interacting with staff, (d) watching, without participating, (e) solitary behavior, or (f) unoccupied behavior. From the data, we created the outcome variable, which represented the percentage of time frames each participant was interacting intergenerationally for each session.

#### BP checklist (predictors)

Observers documented intergenerational activities using the BP Checklist and included the date, the number of child and adult participants and staff, and which BPs were used by activity leaders over the course of the activity (indicated by yes or no responses). A recent factor analysis of BP checklists completed in the current study revealed a 13-item, three-factor solution across intergenerational programming of varied content (Jarrott, Turner et al., 2021). The factor sum scores were used as predictors in our analyses. Factors represent (a) participant pairing, (b) person-centered strategies, and (c) socioemotional accommodations.

##### Factor 1: Promoting participant pairing

This three-item factor involves promoting intergenerational pairs through grouping of persons and materials (e.g., “Seating arrangements used intergenerational pairs or small groups”).

##### Factor 2: Person-centered strategies

Comprised of six items, this factor reflects the concept of personhood (Kitwood, 1997) and concerns activity leaders’ demonstrated respect for and interest in participants’ background, abilities, and preferences (e.g., “Activity leaders discussed the activity in relation to participant interests or experiences to encourage interaction”).

##### Factor 3: Socioemotional accommodations (referred to as staff knowledge of participants in Jarrott, Turner et al., 2021)

This four-item factor reflects the ability of activity leaders, who are primarily responsible for single generation programming, to adapt the setting and respond to participant needs as they pertained to promoting positive intergenerational interactions (e.g., “Adaptations to physical space were made”).

#### Covariates

##### Participant session number

Previous research indicates that participants become more comfortable with intergenerational programming over repeated exposures, which could influence the extent to which they engaged in intergenerational interaction (Gruenewald et al., 2016). Therefore, we created a variable ordering the sessions in chronological order, by date, for each participant to control for potential change over time with “1” being the first session joined by a participant and higher numbers representing participation in later sessions.

##### Phase of program implementation

Program sites joined the study at different time points, and we controlled for the potential influence of timing on BP implementation as we made changes to our training process between phases in response to participant feedback. Phase 1 sites (*n* = 3) joined in the first half of the study, while Phase 2 sites (*n* = 2) joined in the last half of the study. We coded the Phase 1 sessions as 0 and the Phase 2 sessions as 1.

##### Participant demographics

We included participants’ age, race, and gender in the models to account for developmental and demographic characteristics that might influence engagement in intergenerational programming (Femia et al., 2008; Gilbert & Ricketts, 2008; Thompson & Weaver, 2016; Young & Janke, 2013). We treated age as a continuous variable, gender as a categorical dichotomous variable (0 = male, 1 = female), and race as a categorical dichotomous variable based on participants’ indicated membership in up to five categories (0 = White, 1 = non-White).

### Data Analysis

The units of analysis in our study were the participating children and adults. Multilevel modeling captured within-person differences over time, considering the participants as contexts within which BP implementation and intergenerational interaction occurred and varied during the study (Hoffman & Stawski, 2009). We first computed intraclass correlations (ICCs), the measure of how much of a variable’s variance can be attributed to within-person fluctuations over time versus individual differences between participants (Fisher, 1992). ICCs (presented in Table 2) were calculated by dividing the sum of the between-person variation and the within-person variation by the between-person variance. All ICCs were at or below 0.52, indicating nearly or over half of the variability in Factor 1, Factor 2, and Factor 3, and intergenerational interaction was at the within-person level. Taken together, these ICCs provide justification for studying the associations between BP implementation and intergenerational interaction via within-person analysis. Therefore, we ran two separate multilevel models nesting sessions within participants—one model for each of the three factors of the BP Checklist—for both child and adult participants, yielding six total models.

**Table 2.** Descriptive Statistics and Correlations for Study Variables for Children

Variable	n	M	SD	ICC	1.	2.	3.	4.	5.	6.	7.	8.	9.
1. Age	105	3.26	0.66	—	—								
2. Gender	105	—	—	—	0.05	—							
3. Race	105	—	—	—	0.14*	−0.05	—						
4. Participant session number	266	3.75	2.81	—	−0.11	−0.05	−0.09	—					
5. Phase of implementation	266	—	—	—	−0.08	0.17**	−0.35****	0.27****	—				
6. Factor 1^a^	266	2.42	0.78	0.03	0.08	0.15**	−0.04	−0.05	0.15*	—			
7. Factor 2^b^	266	4.71	1.29	0.30	0.10	0.16**	−0.10	−0.10	0.33****	0.25****	—		
8. Factor 3^c^	266	3.55	0.78	0.50	−0.19**	−0.19**	−0.16**	0.16**	0.40****	0.28****	0.15**	—	
9. Intergenerational interaction	266	30.26	27.98	0.22	0.04	0.05	−0.29****	0.12	0.33****	0.27****	0.37****	0.11	—

*Notes:* ICC = intraclass correlation. This table provides descriptive statistics and correlations for intergenerational interaction for children.

^a^Factor 1 scores ranged from 0 to 3.

^b^Factor 2 scores ranged from 1 to 6.

^c^Factor 3 scores ranged from 2 to 4.

*p≤ .05, **p≤ .01, ***p≤ .001, ****p≤ .0001.

To analyze BP implementation at the within-person level, we created person-mean-centered BP implementation (“BP” appended with “mn” in the equations below) from which a session’s difference from the mean was subtracted for each participant on each attended session (Hoffman & Stawski, 2009). Statistical models are as follows, where at Level 1, intergenerational interaction on session *s* for individual *i* is a function of an intercept ($\beta_{0i\;}$), the participant’s session number ($\beta_{1i}$), and the time-varying effect of BP factor implementation ($\beta_{2i}$), which indicates whether session-to-session variation in BP implementation for a participant’s attended sessions systematically was associated with their intergenerational interaction being higher (or better) than usual. $\;\;\;e_{di}$ represents residual. The Level 2 model reflects the between-person differences in Level 1 parameters. As the β _1*i*_ and β _2*i*_ are fixed to be constant in the Level 2 model, the Level 2 model is a random intercept model, where people only differ in their intercepts (initial status), not their slopes. The participant’s age, gender, race, and the phase of implementation are time-invariant variables, so they are put in the Level 2 model to predict the variability of intercept (β _0*i*_). (Across all six models, inclusion of the random effects of the time-varying variables of participant session number failed to converge, were estimated to be 0 [G Matrix not positive definite], or were not significant. Therefore, we only included the intercept as a random effect in the models.)

The models are as follows:

Level 1 (within-person):


$$\begin{array}{ll} \;\;\;IG\;\;\;Interaction_{si\;}= & \;\beta_{0i\;}(Intercept)\\ & +\;\beta_{1i}(Participant\;session\;\;number_{si})\\ & +\;\beta_{2i}(BP\;factor\;implementation_{si}\\ & -BP\;\;factor\;implementationmn_{i})+\;e_{di} \end{array}$$

Level 2 (between-person and random effects):


$$\begin{array}{ll} \beta_{0i\;}= & \;\gamma_{00}+\gamma_{01}(Age_{i})+\;\gamma_{02}(Gender_{i})\\ & +\;\gamma_{03}(Race_{i})\\ & +\;\gamma_{04}(Phase\;of\;\;implementation_{i})+\;U_{0i} \end{array}$$

$$\begin{array}{l} \beta_{1i}=\gamma_{10} \end{array}$$

$$\begin{array}{l} \beta_{2i}=\gamma_{20} \end{array}$$

We conducted analyses with SAS PROC MIXED using maximum likelihood estimation (SAS version 9.04; SAS Institute Inc., Cary, NC).

## Results

Adult participants ranged in age from 55 to 98 years, with an average age of 75.25 years. Among adult participants, 77% were women, and 51% were White. Child participants ranged in age from 2 to 5 years, with an average age of 3.26 years; 51% were female, and 71% were White.

On average, adults participated in 2.79 (range = 1–10) sessions, and children participated in 2.71 sessions (range = 1–11). Each session included an average of 6.72 children and 5.99 adults. The average proportion of children to adults was 1.54, meaning one to two adult participants were present for each child participant, on average. Additional descriptive statistics and Pearson correlations for variables are given in Tables 2 and 3.

**Table 3.** Descriptive Statistics and Correlations for Study Variables for Adults

Variable	n	M	SD	ICC	1.	2.	3.	4.	5.	6.	7.	8.	9.
1. Age	84	75.25	10.53	—	—								
2. Gender	84	—	—	—	0.13	—							
3. Race	84	—	—	—	−0.10	0.22***	—						
4. Participant session number	225	3.63	2.66	—	−0.23**	−0.08	0.03	—					
5. Phase of implementation	225	—	—	—	0.07	−0.24***	−0.40****	0.21***	—				
6. Factor 1^a^	225	2.41	0.81	0.02	−0.01	−0.03	−0.06	0.003	0.09	—			
7. Factor 2^b^	225	4.79	1.29	0.41	0.12	−0.01	−0.19**	−0.002	0.41****	0.23***	—		
8. Factor 3^c^	225	3.51	0.80	0.52	−0.03	−0.25***	−0.24***	0.28****	0.42****	0.26****	0.14*	—	
9. Intergenerational interaction	225	44.43	32.23	0.39	−0.03	−0.01	−0.06	0.17**	0.38****	0.26****	0.36****	0.17**	—

*Notes:* ICC = intraclass correlation. This table provides descriptive statistics and correlations for intergenerational interaction for older adults.

^a^Factor 1 scores ranged from 0 to 3.

^b^Factor 2 scores ranged from 1 to 6.

^c^Factor 3 scores ranged from 2 to 4.

*p≤ .05, **p≤ .01, ***p≤ .001, ****p≤ .0001.

### Factor 1: Promoting Pairing

On days when children participated in a program that had higher Factor 1 BP implementation than the average number of BPs across the children’s sessions, children had higher intergenerational interaction than their own average across all sessions (*B* = 10.26, *SE* = 2.43, *p* < .0001). On days when adults participated in a program that had higher Factor 1 BP implementation than the average number of BPs across the adult’s sessions, adults had higher intergenerational interaction than their own average across all sessions (*B* = 5.95, *SE* = 2.60, *p* = .02). Factor 1 estimates for the full models, including covariates, for child and adult participants are given in Tables 4 and 5, respectively.

**Table 4.** Multilevel Model Parameter Estimates for the Effects of Factor-Level BPs on Intergenerational Interaction for Children

Effect	Parameter	Factor 1 Estimate (*SE*)	Factor 2 Estimate (*SE*)	Factor 3 Estimate (*SE*)
*Fixed effects*				
Intercept	$\beta_{0i\;}$	15.63 (10.40)	15.70 (10.47)	15.41 (10.55)
Age	$\gamma_{01}$	3.36 (2.62)	3.41 (2.64)	3.48 (2.66)
Gender	$\gamma_{02}$	0.64 (3.67)	0.57 (3.69)	0.54 (3.70)
Race	$\gamma_{03}$	−13.55*** (4.10)	−13.52** (4.13)	−13.49** (4.16)
Participant session number	$\;\;\;\beta_{1i}$	0.23 (0.59)	0.20 (0.60)	0.24 (0.61)
Phase of implementation	$\gamma_{04}$	13.69*** (4.12)	13.76*** (4.13)	13.73*** (4.14)
Factor within-person	$\;\;\;\beta_{2i}$	10.26**** (2.43)	5.12** (1.75)	1.93 (3.51)
*Random effects*				
Variance components				
Intercept	$U_{0i}$	80.53* (39.99)	74.26* (39.54)	68.84* (39.21)
Residual	$e_{di}$	545.45**** (55.45)	570.70**** (57.65)	594.33**** (59.72)

*Notes:* BP = best practice. Estimation method: Maximum likelihood.

*p ≤ .05, **p ≤ .01, ***p ≤ .001, ****p ≤ .0001.

**Table 5.** Multilevel Model Parameter Estimates for the Effects of Factor-Level BPs on Intergenerational Interaction for Adults

Effect	Parameter	Factor 1 Estimate (*SE*)	Factor 2 Estimate (*SE*)	Factor 3 Estimate (*SE*)
*Fixed effects*				
Intercept	$\beta_{0i\;}$	40.60* (20.04)	42.28* (20.15)	38.53 (19.95)
Age	$\gamma_{01}$	−0.24 (0.25)	−0.25 (0.25)	−0.22 (0.25)
Gender	$\gamma_{02}$	8.68 (6.65)	8.82 (6.69)	9.58 (6.60)
Race	$\gamma_{03}$	4.47 (5.69)	4.48 (5.72)	4.43 (5.66)
Participant session number	$\;\;\;\beta_{1i}$	0.06 (0.79)	−0.14 (0.78)	0.34 (0.81)
Phase of implementation	$\gamma_{04}$	27.01**** (6.08)	27.16**** (6.13)	26.78**** (6.03)
Factor within-person	$\;\;\;\beta_{2i}$	5.95* (2.60)	7.11*** (2.04)	−3.20 (3.77)
*Random effects*				
Variance components				
Intercept	$U_{0i}$	255.38** (93.29)	272.18** (93.93)	241.70** (92.70)
Residual	$e_{di}$	616.07**** (74.24)	586.78**** (70.79)	638.27**** (76.91)

*Notes:* BP = best practice. Estimation method: Maximum likelihood.

*p ≤ .05, **p ≤ .01, ***p ≤ .001, ****p ≤ .0001.

### Factor 2: Person-Centered Strategies

On days when children participated in a program that had higher Factor 2 BP implementation than the average number of BPs across the children’s sessions, children had higher intergenerational interaction than their own average across all sessions (*B* = 5.12, *SE* = 1.75, *p* = .004). On days when adults participated in a program that had higher Factor 2 BP implementation than the average number of BPs across the adult’s sessions, adults had higher intergenerational interaction than their own average across all sessions (*B* = 7.11, *SE* = 2.04, *p* = .001). Factor 2 estimates for the full models, including covariates, for children and adults are given in Tables 4 and 5, respectively.

### Factor 3: Socioemotional Accommodation

Factor 3 BP implementation did not significantly predict intergenerational interaction for either children (*B* = 1.93, *SE* = 3.51, *p* = .58) or adults (*B* = −3.20, *SE* = 3.77, *p* = .40). Factor 3 estimates for the full models, including covariates, for children and adults are given in Tables 4 and 5, respectively.

### Covariates

Turning to covariates, participant session number was not significantly associated with level of intergenerational interaction. Phase of implementation was significantly associated; child and adult participants at Phase 2 sites exhibited higher levels of interaction than those at Phase 1 sites (Children Factor 1: *B* = 13.01, *SE* = 4.01, *p* < .01; Children Factor 2: *B* = 16.03, *SE* = 3.64, *p* < .0001; Children Factor 3: *B* = 13.68, *SE* = 4.14, *p* < .001; Adult Factor 1: *B* = −27.75, *SE* = 6.08, *p* < .0001; Adult Factor 2: *B* = 29.10, *SE* = 6.10, *p* < .0001; Adult Factor 3: *B* = 28.66, *SE* = 6.05, *p* < .0001). Children whose parents described them as non-White had lower levels of intergenerational interaction than children who were White (Factor 1: *B* = −12.90, *SE* = 4.01, *p* < .001; Factor 2: *B* = −12.95, *SE* = 3.73, *p* < .001; Factor 3: *B* = −13.50, *SE* = 4.15, *p* < .001).

## Discussion

We assessed the association between implementation of BPs during intergenerational programming and intergenerational interaction among children and adults. Through analysis of observational data gathered at five sites over a 4-year period, results indicated that BP implementation by trained activity leaders increased the level of children’s and adults’ intergenerational interaction. Specifically, when activity leaders implemented more practices that paired partners (Factor 1) and were person-centered (Factor 2) during an intergenerational session, individuals achieved higher levels of intergenerational interaction compared to their average level of intergenerational interaction.

Past research supports the major findings of the present study. While a recent scoping review (Jarrott, Scrivano et al., 2021) concluded that most intergenerational research investigating evidence-based practices utilizes qualitative methodology, one quantitative study echoes the importance of facilitator-specific practices. Epstein and Boisvert (2006) describe five practices that promote intergenerational interaction, including a designated programming space, age-appropriate materials, and explicit facilitation by activity leaders. Qualitative data (Jarrott, Scrivano et al., 2021) also point to facilitator-specific practices that affect intergenerational program outcomes, such as setting the environment (Bunting & Lax, 2019), facilitating to promote interaction (Heydon et al., 2017), and incorporating flexibility into activities (Anderson et al., 2017).

BPs reflecting socioemotional accommodation of participants were not significantly associated with levels of participant interaction. Activity leaders may have planned their activities to accommodate participants’ abilities, thereby eliminating the need to adapt to the environment or reduce distractions in a manner that would be noted by the observer during the activity. Other BPs in this factor, like *moving around the activity space* and *responding to both generations of participants*, were implemented in almost 100% of the activities, which limited the factor score range and constricted predictive ability of the factor. If items in this factor are important to participant outcomes, they may need to be emphasized during BP staff training and assessed in a format other than observation during programming.

Participants’ level of intergenerational interaction was also influenced by covariates. Higher levels of interaction were noted among participants attending sites that joined the study after training methods had been revised. We received important feedback from Phase 1 partners and made responsive changes in Phase 2. For example, we shifted from a full-day in-person BP training to a self-paced online training. The shift proved conducive to some activity leaders’ learning styles and schedules. We began sending bi-monthly newsletters to activity leaders and hosted annual project meetings to facilitate continuing education and discussion (Juckett et al., 2021; Weaver et al., 2019). The increased, varied interaction with these Phase 2 activity leaders may have fostered greater comprehension and adoption of BPs.

Our expectation based on previous research that a participant’s level of intergenerational interaction would increase with time as they gained comfort in the intergenerational setting was not met. Rather, both child and adult rates of interaction could be equally high in their first session as in their last session. The results highlight (a) the potential for participants to actively engage with intergenerational partners from the inception of programming and (b) the impact that factors outside of participants (i.e., promoting participant pairs and person-centered strategies by staff) have on their intergenerational engagement. This finding illustrates the importance of activity leaders; those familiar with participants’ interests, abilities, and manner of engaging can develop practices and programming that prepare participants, provide purpose (Owen et al., 2021), and promote success. Results of this analysis are encouraging as intergenerational programs often have revolving participation by design or due to variable program attendance.

Demographics including age, gender, and race can affect participant experiences in intergenerational and other settings (Femia et al., 2008; Gilbert & Rickets, 2008; Gruenewald et al., 2016; Young & Janke, 2013); therefore, we included these items as covariates. Neither age nor gender was associated with levels of intergenerational interaction, which may encourage activity leaders to develop inclusive programming that will appeal to diverse participants (i.e., more men). While extant literature has associated race with adults’ but not child participants’ intergenerational program experiences (Young & Janke, 2013), our data revealed that children whose parents described them as non-White race experienced significantly lower levels of intergenerational interaction than White children. Children’s comfort may have been lower when interacting with adults belonging to a different racial group, but the proportion of non-White and White adults and children was comparable within sites.

### Strengths and Limitations

Our findings offer a useful contribution to the field as practitioners seek resources to train activity leaders and measure the impact of intergenerational programming on participants (Jarrott & Lee, in press). Taken together with other analyses of this data set (Jarrott, Turner et al., 2021; Juckett et al., 2021; Weaver et al., 2019), we refined the BP Checklist (Jarrott et al., 2019) and crafted a complimentary continuing education course (Jarrott, Juris et al., 2021) that supports activity leaders’ ability to adopt evidence-based practices. Our work builds upon and is supported by other intergenerational leaders (e.g., Generations United; Generations Working Together) who champion thoughtful practice grounded in evidence for intergenerational program success.

The methodology we used in the current study carries both strengths and weaknesses. This is the first study to utilize an intraindividual variability approach to analyze how intergenerational program facilitation influences intergenerational engagement among child and adult participants. Employing such a method allowed us to capture the session-by-session nuance present in our data and likely present in other intergenerational programs. However, there is no *de facto* approach for determining sample size for multilevel models; thus, it is possible that our sample size of 105 children and 84 adults with varying numbers of sessions attended could be too small to render our results conclusive.

Intergenerational practitioners employ a wide array of programming content to achieve identified goals; therefore, we developed the BPs to apply across assorted program content. We view the application of BPs to diverse program content as a strength of the training and evaluation tools presented in our study. However, with content varying across sessions both within and between sites, generalizability of findings may be affected by heterogeneous program content.

While observers were trained to code participants’ and activity leaders’ behaviors, the method for training and checking the reliability of BP coding lacked the rigor of training protocol for the IOS. The IOS training protocol was established before the study began, but early adaptations to the BP Checklist based on activity leader input necessitated adapted training strategies and checks of reliability for observers. Future studies should adopt a standardized protocol for training and establishing reliability using the BP Checklist. Nonetheless, our results offer a first step in analyzing how intergenerational program BP implementation affects participant engagement at the within-participant level.

### Future Research

Both children and adults were highly variable in the extent to which they engaged in intergenerational interaction as indicated in the ICCs. Our analyses demonstrate that some, but not all, of this variability can be attributed to activity leader implementation of BPs. Further work should explore what other factors cause session-by-session variation. For example, some practitioners form routine partnerships in which the same child and adult pair for every session. While our data reflected opportunities for one-to-one partnerships, we could not determine if children and adults had consistent partners across sessions. Variability in programming content could also contribute to fluctuating responses of participants, which could be held constant in future studies. Some variability between sessions is to be expected; however, if it negatively affects the outcomes of intergenerational programming, additional practices may need to be identified to promote consistently high levels of intergenerational interaction.

Given our unexpected findings that session number did not influence participants’ level of intergenerational interaction, it would be interesting to explore other outcomes that may reflect the impact of sustained participation. While programming in the current study consisted of discrete activities with variable participation, other intergenerational programs are designed to achieve outcomes through repeated engagement with program content (Gruenewald, 2016). Using the BP Checklist and IOS in conjunction with such an intergenerational program might reveal that the number of sessions a participant joins (as a proxy for dosage) influences participant outcomes. For example, researchers of intergenerational co-mentoring used to achieve improved mental health among vulnerable children might adopt the BP Checklist, the IOS, and measures of life skills and find an association between the number of sessions a participant joins and their life skills outcomes. Similarly, our finding that intergenerational interaction can be high on the first session suggests that short-term programs offer some merit depending on desired outcomes.

Children from diverse backgrounds are likely taught various expectations for engaging with older adults (Gilbert & Rickets, 2008). With increasing attention to diversity, equity, and inclusion training for educators (Fuentes et al., 2021), further understanding of how children engage in diverse intergenerational settings is an area for future intergenerational research.

## Conclusions

Intergenerational programs aim to achieve a wide range of goals for younger and older participants, primarily by facilitating positive intergenerational interactions. Theory is widely used to guide practice that facilitates conditions for positive intergroup exchange, but the practices that promote or deter these exchanges are rarely measured. Thus, the mechanisms by which achievement of program goals, such as participants’ attitudes, knowledge, and skills remain largely unknown. The BP Checklist, informed by theories that drive many intergenerational programs (Allport, 1954; Erikson, 1982; Kitwood, 1997; Pettigrew, 1998; Vygotsky, 1978), demonstrates utility in explaining within-person differences among both child and adult participants. Findings from this observational study equip intergenerational activity leaders with easy-to-implement strategies of promoting pairing and focusing on specific person-centered strategies to increase intergenerational interaction. Intergenerational relationships may be powerful means to achieve diverse goals—they depend on skillful practice by trained activity leaders.
